# Reliability of Trial Information Across Registries for Trials With Multiple Registrations: A Systematic Review

**DOI:** 10.1001/jamanetworkopen.2021.28898

**Published:** 2021-11-01

**Authors:** Benjamin Speich, Viktoria L. Gloy, Katharina Klatte, Dmitry Gryaznov, Ala Taji Heravi, Nilabh Ghosh, Ioana R. Marian, Hopin Lee, Anita Mansouri, Szimonetta Lohner, Ramon Saccilotto, Edris Nury, An-Wen Chan, Anette Blümle, Ayodele Odutayo, Sally Hopewell, Matthias Briel

**Affiliations:** 1 Basel Institute for Clinical Epidemiology and Biostatistics, Department of Clinical Research, University Hospital Basel, University of Basel, Basel, Switzerland; 2 Centre for Statistics in Medicine, Nuffield Department of Orthopaedics, Rheumatology and Musculoskeletal Sciences, University of Oxford, Oxford, United Kingdom; 3 Clinical Trial Unit, Department of Clinical Research, University Hospital Basel, University of Basel, Basel, Switzerland; 4 Swiss Tropical and Public Health Institute, Basel, Switzerland; 5 Department of Neurosurgery and Department of Biomedicine, University Hospital Basel, University of Basel, Basel, Switzerland; 6 Oxford Clinical Trials Research Unit, Centre for Statistics in Medicine, Nuffield Department of Orthopaedics, Rheumatology and Musculoskeletal Sciences, University of Oxford, Oxford, United Kingdom; 7 Cochrane Hungary, Clinical Centre of the University of Pécs, Medical School, University of Pécs, Pécs, Hungary; 8 Institute for Evidence in Medicine (for Cochrane Germany Foundation), Faculty of Medicine and Medical Center, University of Freiburg, Freiburg, Germany; 9 Department of Medicine, Women’s College Research Institute, Women’s College Hospital, University of Toronto, Toronto, Ontario, Canada; 10 Applied Health Research Centre, Li Ka Shing Knowledge Institute of St Michael's Hospital, Toronto, Ontario, Canada; 11 Department of Health Research Methods, Evidence, and Impact, McMaster University, Hamilton, Ontario, Canada

## Abstract

**Importance:**

Clinical trial registries are important for gaining an overview of ongoing research efforts and for deterring and identifying publication bias and selective outcome reporting. The reliability of the information in trial registries is uncertain.

**Objective:**

To assess the reliability of information across registries for trials with multiple registrations.

**Evidence Review:**

For this systematic review, 360 protocols of randomized clinical trials (RCTs) approved by research ethics committees in Switzerland, the UK, Canada, and Germany in 2012 were evaluated. Clinical trial registries were searched from March to September 2019 for corresponding registrations of these RCTs. For RCTS that were recorded in more than 1 clinical trial registry, key trial characteristics that should be identical among all trial registries (ie, sponsor, funding source, primary outcome, target sample size, trial status, date of first patient enrollment, results available, and main publication indexed) were extracted in duplicate. Agreement between the different trial registries for these key characteristics was analyzed descriptively. Data analyses were conducted from May 1 to November 30, 2020. Representatives from clinical trial registries were interviewed to discuss the study findings between February 1 and March 31, 2021.

**Findings:**

The analysis included 197 RCTs registered in more than 1 trial registry (151 in 2 registries and 46 in 3 registries), with 188 trials in ClinicalTrials.gov, 185 in the European Union Drug Regulating Authorities Clinical Trials Database (EudraCT), 20 in ISRCTN, and 47 in other registries. The agreement of key information across all registries was as follows: 178 of 197 RCTs (90%; 95% CI, 85%-94%) for sponsor, 18 of 20 (90%; 95% CI, 68%-99%) for funding source (funding was not reported on ClinicalTrials.gov), 154 of 197 (78%; 95% CI, 72%-84%) for primary outcome, 90 of 197 (46%; 95% CI, 39%-53%) for trial status, 122 of 194 (63%; 95% CI, 56%-70%) for target sample size, and 43 of 57 (75%; 95% CI, 62%-86%) for the date of first patient enrollment when the comparison time was increased to 30 days (date of first patient enrollment was not reported on EudraCT). For results availability in trial registries, agreement was 122 of 197 RCTs (62%; 95% CI, 55%-69%) for summary data reported in the registry and 91 of 197 (46%; 95% CI, 39%-53%) for whether a published article with the main results was indexed. Different legal requirements were stated as the main reason for inconsistencies by representatives of clinical trial registries.

**Conclusions and Relevance:**

In this systematic review, for a substantial proportion of registered RCTs, information about key trial characteristics was inconsistent across trial registries, raising concerns about the reliability of the information provided in these registries. Further harmonization across clinical trial registries may be necessary to increase their usefulness.

## Key Points

**Question:**

Are clinical trial registry data for trials with multiple registrations reliable?

**Findings:**

In this systematic review of 197 randomized clinical trials registered in more than 1 trial registry, sponsor and funder had the highest agreement level (90%) among registries. Primary outcome had agreement of 78%; trial status, 46%; and target sample size, 63%.

**Meaning:**

The findings suggest that there is low reliability of key characteristics in clinical trial registries, raising concerns about the usefulness of the information provided in the registries.

## Teaser

This systematic review assesses the consistency of information across registries for clinical trials with multiple registrations.

## Introduction

Randomized clinical trials (RCTs) are at the top of classical textbook evidence-based medicine pyramids.^1,2^ However, RCTs reach their full potential only if they are designed, conducted, and reported appropriately. In an article in 2003, Dickersin and Rennie^3^ highlighted the lack of transparent overview of clinical research and concluded that “a comprehensive register of initiated clinical trials, with each trial assigned a unique identifier, would inform reviewers, physicians, and others (eg, consumers) about which trials had been started and directly address the problem of publication bias.” In 2005, the International Committee of Medical Journal Editors^4^ reported that newly implemented trials (ie, those started after July 2005) would be considered for publication only if they were prospectively registered before enrollment of the first trial participant.

At present, clinical trial registries are well established and are supposed to provide a comprehensive overview of all ongoing RCTs on a specific topic to help deter unnecessary duplication of research and to estimate and deter publication bias.^5^ Furthermore, outcomes must be prespecified in clinical trial registries, which should discourage authors from cherry-picking results in RCTs.^6,7^ Therefore, clinical trial registries can be used as a tool to assess whether outcomes are reported in the final published article as previously specified or whether the primary outcome was changed and new outcomes were introduced.^8,9^ The ongoing COVID-19 pandemic has further underlined the importance of clinical trial registries for providing an overview of ongoing research efforts and, thus, for creating synergies.^10,11,12^ Nevertheless, the reliability of the information found on clinical trial registration websites remains uncertain.^13^ Because some trials are registered in more than 1 clinical trial registry (eg, owing to different requirements by the sponsor and funder or by different participating countries), we empirically assessed the reliability of information on RCTs available in multiple clinical trial registries.

## Methods

### Search and Data Extraction

For this systematic review, we used data from a previous study^14^ on 360 RCT protocols (excluding pilot and phase 1 studies) that were approved by research ethics committees in 2012 in Switzerland, the UK, Germany, and Canada. We searched for registration records of each of these RCTs from March to 2019 using the following platforms: the World Health Organization (WHO) International Clinical Trials Registry Platform, ClinicalTrials.gov, the European Union Drug Regulating Authorities Clinical Trials Database (EudraCT), the ISRCTN registry, and the Google web search engine. We used registration numbers provided in study protocols (when available) or combinations of the population, intervention, control, or primary outcome as search terms together with the name of the principal investigator. Other clinical trial platforms (eg, German Clinical Trials Register, Clinical Trials Registry–India) were considered if they were identified through the aforementioned search platforms (ie, WHO search portal or Google web search). This study followed the Preferred Reporting Items for Systematic Reviews and Meta Analyses (PRISMA) guideline.^15^

When an RCT was identified in a trial registry, we extracted key trial characteristics (ie, sponsor, funding source, primary outcome, target sample size, recruitment status, and date of first patient enrollment). The key trial characteristics were selected from the WHO Trial Registration Data Set,^16^ excluding items that could or should be different among different registries (eg, registration number, date of registration, and contact for public queries) and items that would have required substantially more resources to conduct a meaningful comparison (eg, inclusion and exclusion criteria, secondary outcomes). Furthermore, we checked whether the results of the study and a link to the main published article (containing primary results) were available in the registry records. The searches and data extraction were performed in duplicate by some of us (B.S., V.L.G., K.K., A.T.H., N.G., I.R.M., H.L., A.M., S.L., E.N., and A.B.), and disagreements were resolved by discussion.

### Data Analysis

Data analyses were conducted from May 1 to November 30, 2020. To assess the reliability of the trial information, we analyzed whether the key trial characteristics were identical in all identified trial registries. Furthermore, we evaluated, in a 1-to-1 comparison of registries, the agreement between the information available in the most commonly identified primary clinical trial registries (ie, ClinicalTrials.gov vs EudraCT, ClinicalTrials.gov vs other registries, and EudraCT vs other registries). Because clinical trial registries used different terms to describe trial status, the following categories were created: (1) completed, (2) ongoing (with or without recruitment), (3) terminated, and (4) unclear. The trial status on EudraCT was reported by each participating country separately. If the status was recorded as ongoing in any participating country, we judged the overall status to be ongoing for the study. When some countries stated that the trial was terminated and others indicated that it was completed, we judged the status to be unclear. For the variable date of first patient enrolled, we added an additional analysis to assess whether the indicated date was in agreement among the different registries when the comparison time was increased to 30 days. For comparison of the primary outcomes, we compared the time point at which the outcome was measured and the analysis metric (eg, change from baseline, final values) in addition to the type of outcome. We judged the primary outcome to be “potentially identical, but some details missing in 1 registry,” if 1 of these outcome characteristics was reported in 1 registry but missing from another. We conducted a stratified analysis to explore whether there was a difference in registry agreement between industry-sponsored and investigator-sponsored RCTs. The sample of analyzed studies was reduced for a few trial characteristics that were not systematically reported in some registries (ie, funding source and date when first patient was enrolled). In response to peer-review feedback, we compared the primary outcome and target sample size for RCTs with discrepancies among trial registries with those reported in the published article with the main results. All analyses were conducted using Stata, version 16.1 (StataCorp LLC) and were descriptive (including 95% CIs) without any formal hypothesis testing.

### Interviews With Clinical Trial Registry Representatives

Representatives from the 7 trial registries (ClinicalTrials.gov, EudraCT, ISRCTN registry, the German Clinical Trials Register, the Clinical Trials Registry–India, the Australian New Zealand Clinical Trials Registry, the Japan Primary Registries Network) included in this study were contacted by email. We presented our results and invited them to a short interview to discuss the following key points: (1) Do you plan to work toward more harmonization of registry items across registries (eg, plan to add items for trial characteristics that are present in other registries)? (2) Do you know of any developments since 2012 that could have improved the reliability of information available in clinical trial registries? (3) What measures could be undertaken to improve the agreement of clinical trial registries? Could other stakeholders, such as funding agencies, research ethics committees, or academic institutions, have a role here? and (4) Are you aware of other studies that investigated the consistency of RCT information across registries? Interviews were recorded with the oral permission of clinical trial registry representatives. Answers were summarized qualitatively, focusing on the 4 aforementioned key points.

## Results

### Reliability of Available Information in Clinical Trial Registries

From the sample of 360 RCT protocols approved in 2012, 197 were registered in more than 1 clinical trial registry. Of those 197 RCTs, 151 (77%) were identified in 2 registries and 46 (23%) in 3 registries (Table 1). The RCTs were registered in the following clinical trial registries: ClinicalTrials.gov (188 [95%]), EudraCT (185 [94%]), ISRCTN registry (20 [10%]), and other registries (47 [24%]), including the German Clinical Trials Register (n = 33), the Clinical Trials Registry–India (n = 11), the Australian New Zealand Clinical Trials Registry (n = 2), and the Japan Primary Registries Network (n = 1). The RCT protocols were originally approved in Switzerland (92 [47%]), the UK (58 [29%]), Germany (29 [15%]), and Canada (18 [9%]). Of the 197 trials, 171 (87%) included multiple centers, and 155 (79%) were industry sponsored.

**Table 1.** Characteristics of 197 Randomized Clinical Trials That Received Ethical Approval in 2012 and Were Registered in More Than 1 Clinical Trial Registry

Characteristic	Trials, No. (%)
Identified registry entries, No.	
2	151 (77)
3	46 (23)
Sponsor	
Industry	155 (79)
Investigator	42 (21)
Center status	
Single center	8 (4)
Multicenter	171 (87)
Unclear	18 (9)
Clinical trial registry	
ClinicalTrials.gov	189 (96)
EudraCT	185 (94)
ISRCTN	20 (10)
Other registries^a^	47 (24)
Country of ethics approval	
Switzerland	92 (47)
UK	58 (29)
Germany	29 (15)
Canada	18 (9)

Abbreviation: EudraCT, European Union Drug Regulating Authorities Clinical Trials Database.

^a^ German Clinical Trials Register (n = 33), Clinical Trials Registry–India (n = 11), Australian New Zealand Clinical Trials Registry (n = 2), Japan Primary Registries Network (n = 1).

Most of the variables of interest were well reported (ie, >90%) in the clinical trial registries (Table 2). The 2 exceptions were date of first patient enrollment, which was not reported on EudraCT, and funding source, which was in a merged entry field on ClinicalTrials.gov under “Collaborators” and was also not consistently available on EudraCT (165 of 185 RCTs [89%]). The main publication was indexed in 101 of 188 ClinicalTrials.gov registrations (54%), 8 of 20 ISRCTN registrations (40%), 15 of 185 EudraCT registrations (8%), and 2 of 47 other registrations (4%). Results were posted on 127 of 185 EudraCT registrations (69%) and 107 of 188 ClinicalTrials.gov registrations (57%) but on none of the ISRCTN or other registrations (Table 2).

**Table 2.** Variables, Results, and Links to Published Articles Reported in the Clinical Trial Registries

Variable	ClinicalTrials.gov	EudraCT	ISRCTN	Other registries^a^
Identified RCTs, No.	188	185	20	47
Reported variables, No. (%)				
Primary outcome	188 (100)	184 (99)	20 (100)	46 (98)
Target sample size	186 (99)	185 (100)	20 (100)	44 (94)
Date of first patient enrollment	188 (100)	0^b^	20 (100)	46 (98)
Funding source	2 (1)^c^	165 (89)	20 (100)	47 (100)
Sponsor	188 (100)	185 (100)	20 (100)	47 (100)
Status of study	188 (100)	185 (100)	20 (100)	47 (100)
Link to published article, No (%)	101 (54)	15 (8)	8 (40)	2 (4)
Results posted, No. (%)	107 (57)	127 (69)	0	0

Abbreviations: EudraCT, European Union Drug Regulating Authorities Clinical Trials Database; RCT, randomized clinical trial.

^a^ German Clinical Trials Register (n = 33); Clinical Trials Registry–India (n = 11); Australian New Zealand Clinical Trials Registry (n = 2); Japan Primary Registries Network (n = 1).

^b^ EudraCT did not report the date of the first patient enrolled.

^c^ ClinicalTrials.gov did not have a separate field to enter the funder of the study; 2 RCTs reported this separately in the “More Information” section.

The proportion of trials with consistent information across all registries was 178 of 197 (90%; 95% CI, 85%-94%) for sponsor, 18 of 20 (90%; 95% CI, 68%-99%) for funding source (funding was not reported on ClinicalTrials.gov), and 154 of 197 (78%; 95% CI, 72%-84%) for the primary outcome (Table 3). The primary outcomes of another 17 of 197 RCTs (9%; 95% CI, 5%-13%) were potentially identical, but some key characteristics (eg, time point of measurement or assessment) were missing in at least 1 registry. Furthermore, the agreement across all registries was 90 of 197 RCTs (46%; 95% CI, 39%-53%) for the trial status, 122 of 194 (63%; 95% CI, 39%-53%) for the target sample size, and 1 of 57 (2%; 95% CI, 0%-9%) for the date of first patient enrollment (date of first patient enrollment was not reported on EudraCT). When the time was increased to 30 days, the agreement for date of first patient enrollment increased to 43 of 57 (75%; 95% CI, 62%-86%). The results from the 1-to-1 comparison were in line with the comparison across all registries (Table 3).

**Table 3.** Reliability of Clinical Trial Registries Measured as Agreement for Specific Trial Characteristics Across Different Trial Registries

Characteristic	All registries		ClinicalTrials.gov vs EudraCT		ClinicalTrials.gov vs other registries^a^		EudraCT vs other registries^a^	
	No./total No.	% (95% CI)	No./total No.	% (95% CI)	No./total No.	% (95% CI)	No./total No.	% (95% CI)
Sponsor identical	178/197	90 (85-94)	161/177	91 (86-95)	43/46	93 (82-99)	39/42	93 (81-99)
Primary outcome								
Identical	154/197	78 (72-84)	142/177	80 (74-86)	37/46	80 (66-91)	34/42	81 (66-91)
Potentially identical but some details missing in 1 registry	17/197	9 (5-13)	14/177	8 (4-13)	2/46	4 (1-15)	3/42	7 (1-19)
Different	25/197	13 (8-18)	20/177	11 (7-17)	6/46	13 (5-26)	4/42	10 (3-23)
Missing in at least 1	1/197	1 (0-3)	1/177	1 (0-3)	1/46	2 (0-12)	1/42	2 (0-13)
Identical status of trial^b^	90/197	46 (39-53)	88/177	50 (42-57)	25/46	54 (39-69)	15/42	35 (22-52)
Identical target sample size	122/194	63 (56-70)	113/176	64 (57-71)	33/43	77 (61-88)	28/40	70 (53-83)
Identical funding source	18/20	90 (68-99)	NA	NA	1/1	100	8/8	100
Date first patient enrolled identical	1/57	2 (0-9)	NA	NA	0/45	0	NA	NA
Date first patient enrolled identical after 30 d	43/57	75 (62-86)	NA	NA	37/45	82 (68-92)	NA	NA
Results								
Available in registry	122/197	62 (55-69)	135/177	76 (69-82)	19/46	41 (27-57)	11/42	26 (14-42)
Available for only RCTs considered with results in ≥1 registry	67/142	47 (39-56)	92/134	69 (60-76)	0/27	0	0/31	0
Main publication listed in registry	91/197	46 (39-53)	86/177	49 (41-56)	24/46	52 (37-67)	38/42	90 (77-97)
Main publication listed for only RCTs considered when results indexed in ≥1 registry	11/114	10 (5-17)	8/99	8 (4-15)	0/19	0	0/3	0

Abbreviations: EudraCT, European Union Drug Regulating Authorities Clinical Trials Database; NA, not applicable; RCT, randomized clinical trial.

^a^ German Clinical Trials Register (n = 33); Clinical Trials Registry–India (n = 11); Australian New Zealand Clinical Trials Registry (n = 2); Japan Primary Registries Network (n = 1).

^b^ EudraCT reports the status per participating countries. If the status was listed as ongoing in any country, we judged the overall status to be ongoing for the study. For 6 studies, the status in some countries was listed as completed, whereas in other countries, it was listed as discontinued. For those 6 studies, we judged the status to be unknown.

The agreement across all registries with respect to results availability was 122 of 197 RCTs (62%; 95% CI, 55%-69%) for summary data reported in the registry and 91 of 197 (46%; 95% CI, 39%-53%) for whether a published article reporting the main results was listed. When we considered only RCTs with results available in at least 1 registry (ie, excluding RCTs without any results in any registry), the agreement decreased to 67 of 142 (47%; 95% CI, 39%-53%). For indexed publications, the agreement decreased to 11 of 114 (10%; 95% CI, 5%-17%) when we only considered RCTs with a publication indexed in at least 1 registry. These findings appeared to be consistent between subgroups of industry-sponsored and investigator-sponsored trials (Table 4). When trials with inconsistent registry entries were assessed for sample size or primary outcome, the agreement between the information reported in the publication and that reported in trial registries was low to moderate for all trial registries (Table 5).

**Table 4.** Analysis Stratified by Sponsor Assessing the Reliability of Clinical Trial Registries

Characteristic	Industry-sponsored RCTs (n = 155)		Investigator-sponsored RCTs (n = 42)	
	No./total No.	% (95% CI)	No./total No.	% (95% CI)
Sponsor identical	146/155	94 (89-97)	32/42	76 (61-88)
Primary outcome				
Identical	122/155	79 (71-85)	32/42	76 (61-88)
Potentially identical but some details missing in 1 registry	13/155	8 (5-14)	4/42	10 (3-23)
Different	20/155	13 (8-19)	6/42	14 (5-29)
Missing in at least 1	1/155	1 (0-4)	NA	NA
Identical status of trial^a^	66/155	43 (35-51)	24/42	57 (41-72)
Identical target sample size	90/153	59 (51-67)	32/41	78 (62-89)
Identical funding source	9/9	100	9/11	82 (48-98)
Date first patient enrolled identical	0/41	0	1/16	6 (0-30)
Date first patient enrolled identical after 30 d	33/41	80 (65-91)	10/16	63 (35-85)
Results				
Available in registry	88/155	57 (49-65)	34/42	81 (66-91)
Available for only RCTs considered with results in ≥1 registry	67/134	50 (41-59)	0/8	0
Main publication listed in registry	62/155	40 (32-48)	29/42	69 (53-82)
Main publication listed for only RCTs considered when results indexed in ≥1 registry	5/97	5 (1-12)	6/17	35 (14-62)

Abbreviations: EudraCT, European Union Drug Regulating Authorities Clinical Trials Database; RCT, randomized clinical trial.

^a^ EudraCT reports the status per participating countries. If the status was listed as ongoing in any country, we judged the overall status to be ongoing for the study. For 6 studies, the status in some countries was listed as completed, whereas in other countries, it was listed as discontinued. For those 6 studies, we judged the status to be unknown.

**Table 5.** Agreement Between Information in the Registry and Information Reported in the Published Trials Across Registries

Variable	ClinicalTrials.gov		EudraCT		ISRCTN		Other registries^a^	
	No./total No.	% (95% CI)	No./total No.	% (95% CI)	No./total No.	% (95% CI)	No./total No.	% (95% CI)
Different primary outcome (n = 25)^a^	9/14	64 (35-87)	6/14	43 (18-71)	1/3	33 (1-91)	1/5	20 (1-72)
Different planned sample size (n = 75)^b^	16/46	35 (21-50)	16/40	40 (25-57)	2/5	40 (5-85)	4/8	50 (16-84)

Abbreviations: EudraCT, European Union Drug Regulating Authorities Clinical Trials Database; RCT randomized clinical trial.

^a^ No publication identified for 6 RCTs, 1 published article did not define primary outcome, and 1 explained different registry entries.

^b^ No publication identified for 13 RCTs and no planned sample size reported in 16 published articles.

### Perspectives of Clinical Trial Registry Representatives

Representatives from ClinicalTrials.gov, EudraCT, ISRCTN, the German Clinical Trials Register, and the Australian New Zealand Clinical Trials Registry agreed to participate in a short interview from February 1 to March 31, 2021. The representatives reported that there were ongoing efforts to harmonize clinical trial registries, referring to the implemented standardized WHO Trial Registration Data Set^16^ and to regular meetings organized by the WHO, as well as bilateral meetings among trial registry representatives. Even though these efforts have been made and continue, different legislation requirements were mentioned as the main hurdle to achieve better harmonization among trial registries. One representative stated, “The main challenge is that even when there is a desire to harmonize, each region’s legal requirements take priority and can’t easily be changed.” Another stated, “Although sponsors are required by law to submit information in our database that is in line with the content of their trials’ protocols and results, there is no legal basis that would require sponsors to submit the same information as it was previously submitted in other databases.” All representatives assumed that lack of regular updates to all trial registries was the main reason for the inconsistencies among trial registries. Representatives emphasized that it is the responsibility of the study sponsor to make sure that the information in study registries is correct and up to date. With regard to some initiatives that might have improved the situation, most representatives mentioned that regular reminders were implemented not only to add study results but also to update trial characteristics. One representative commented, “If you don’t ask them, they don’t tell you about the changes.” In addition to the option to upload study results (possible for all registries but introduced after 2012 for some registries), most registries provide the option to upload study protocols, which could help to ensure that important information, such as the primary outcome, remains consistent among different sources.

When asked what other stakeholders could contribute to improve the situation, 2 representatives mentioned independently that journals should do some basic checks, such as assessing whether the registration actually exists. One representative stated, “We see that publications exist with a provisional registration number but the registration is not publicly available.” Another representative stated that better practices in clinical trials need to be promoted at all levels: “We hear that primary outcomes aren’t even well specified in the primary protocol document itself. So it’s really difficult to have a well-defined primary outcome on the registry when you are working with some material that is suboptimal.” In addition, it was mentioned that it would be good to link final reports from research ethics committees with trial registries. None of the representatives were aware of a similar study assessing the agreement of study information across registries.

## Discussion

Clinical trial registries were established to identify and deter publication bias and selective reporting of outcomes as well as to provide an overview of ongoing research efforts. To fulfill this purpose, the information in clinical trial registries should be reliable.^13^ Although the WHO states that “Trials should only be included on more than 1 registry if it is absolutely necessary,”^17^ country-specific requirements often force investigators to register a trial in multiple trial registries (ie, multinational studies). We used the availability of multiple trial registrations to assess the agreement of information in clinical trial registries. Our study findings suggest that the consistency of key information across registries may be variable for a given trial. For none of the examined characteristics was the agreement above 90%. Only 2 characteristics, sponsor and funder, reached an agreement level of 90%. For all other characteristics, the agreement was lower. This is especially concerning for important key characteristics, such as primary outcome (78% agreement), trial status (46% agreement), and target sample size (63% agreement). The differences in regard to completeness of study results in trial registries may be attributable to some registries having introduced the option to upload study results after 2012; also, listing within the registry whether the study results were published has been mandatory only since 2018.^18^ Interviews with representatives from clinical trial registries showed that although some efforts to harmonize registries have been made, these efforts have mainly focused on the presentation of study results rather than on trial characteristics. In addition, interviewees suggested that different legal requirements in different countries hamper the harmonization of trial registries. Although several studies^8,19,20^ have revealed that there may be large discrepancies between information reported in a clinical trial registry and information that is later published, this is, to our knowledge, the first investigation assessing the consistency of records across trial registries for the same trial.

### Limitations

This study has limitations. First, the sample consisted mostly of multicenter industry trials, and the size is relatively small, considering the vast amount of registered RCTs; therefore, it might not be fully representative. This finding raises the question of whether investigator-initiated trials or single-center trials might perform better. Our subgroup analysis suggested that the performance of investigator-sponsored trials was equally poor. This finding is in line with several research studies^21,22^ that found that industry trials and multicenter trials often performed similarly or even better when adequate reporting was assessed (eg, publishing study results; adherence to Consolidated Standards of Reporting Trials [CONSORT] guidelines). Second, the sample consisted of RCTs that received ethical approval in 2012. The situation may have changed since then, and characteristics of RCTs that are currently registered may have become more reliable. For example, as mentioned by the representatives of trial registries in the interviews, some registries have started to facilitate uploading of trial results (ie, ISRCTN, EudraCT, and the Australian New Zealand Clinical Trials Registry) and introduced or will introduce more user-friendly platforms (EudraCT, the German Clinical Trials Register); others started sending reminders about the trial status (ClinicaTrial.gov, the Australian New Zealand Clinical Trials Registry) or have restructured how primary endpoints should be reported after a study revealed that the reporting quality was relatively poor (ClinicalTrials.gov).^23,24^ However, as Tse et al^24^ stated when discussing the quality of trial registry information, “quality control review cannot ensure the veracity of the submitted information.” Other practices, such as uploading the study protocol, might be more beneficial for the agreement of trial information across registries. This practice was partially implemented in ClinicalTrials.gov, where since 2017, the trial results reported on the registry have been required to include the trial protocol (optional during registration). Third, we extracted data from multiple registries that were available on the day of extraction and did not consider older available versions of registry entries. It is likely that discrepancies arose because some registries were updated while others were not. From a user’s perspective, we believe that this is irrelevant and therefore not a real limitation because a user (eg, scientist or patient) should be able to trust the information of a trial registry at any given time point. Fourth, investigators may have entered country-specific values for some characteristics (eg, sample size, recruitment of first patient). We believe that this was not likely because most definitions of trial characteristics clearly stated that data for the overall trial were required (eg, “Planned number of subjects to be included in the whole clinical trial,” as defined on EudraCT). Entering of country-specific data would be misleading from a user’s perspective because none of the trial characteristics were labeled as being country specific. Fifth, assessment of which registry was most accurate could be done only in a limited scope on a small sample in this study (Table 5). Larger studies would be required to make a meaningful direct comparison among trial registries.

## Conclusions

In this systematic review, for a substantial proportion of registered RCTs, information about key trial characteristics was inconsistent across trial registries, raising concerns about the reliability of clinical trial registries. Further investigation and harmonization efforts across clinical trial registries appear to be necessary to increase their usefulness. Interventions that might increase the reliability include uploading of study protocols (entire protocols or key parts) or linking trial registries through platforms (eg, WHO International Clinical Trials Registry Platform) and implementing automated systems that detect inconsistencies. These 2 interventions may help avoid differences in characteristics that are specified before the start of the trial (ie, uploading trial protocol to specify sponsor, funder, target sample size, and primary outcome) and differences that could be introduced during the course of the trial (ie, actively comparing entries across registries for recruitment of first patient, trial status, and availability of study results and providing links to publication of main results).

## Notes

Supplement. **Nonauthor Collaborators.** Adherence to Spirit Recommendations (ASPIRE) Study Group
