# Supplementary material for: Reliability of Trial Information Across Registries for Trials With Multiple Registrations: A Systematic Review
Source: JAMA Netw Open. 2021 Nov 1;4(11):e2128898. doi: 10.1001/jamanetworkopen.2021.28898 (PMC8561329; doi:10.1001/jamanetworkopen.2021.28898)
Supplement: Supplement. — Nonauthor Collaborators. Adherence to Spirit Recommendations (ASPIRE) Study Group [file jamanetwopen-e2128898-s001.pdf]

Supplemental Online Content: Nonauthor Collaborators

*Indicates required information. Only first name, last name, and suffix will appear in PubMed.

*Group Name(s): Adherence to Spirit Recommendations (ASPIRE) Study Group									
***First Name and Mi**	***Last Name**	***Suffix (eg**	Academic [	Institution	Location (city, state/province, country)	Role or Contribution, eg, chair, principal investigator	Group (if more than 1 Group listed in the byline) and/or Subgroup (eg, Steering Committee)		
Benjamin	Speich		PhD	Basel Institute for Clinical Epidemiology and Biostatistics, Department of Clinical Research, University Hospital Basel, University of Basel	Basel, Switzerland	Designed the study, systematically searched for the trials in clinical trial registries and extracted the trial characteristics, conducted interviews with representatives from trial registries, analyzed the data and wrote the first draft of the manuscript, critically revised the manuscript and approved the final version.			
Viktoria L	Gloy		PhD	Basel Institute for Clinical Epidemiology and Biostatistics, Department of Clinical Research, University Hospital Basel, University of Basel	Basel, Switzerland	Systematically searched for the trials in clinical trial registries and extracted the trial characteristics, critically revised the manuscript and approved the final version.			

Supplemental Online Content: Nonauthor Collaborators

*Indicates required information. Only first name, last name, and suffix will appear in PubMed.

***First Name and Mi**	***Last Name**	***Suffix (eg**	Academic [	Institution	Location (city, state/province, country)	Role or Contribution, eg, chair, principal investigator	Group (if more than 1 Group listed in the byline) and/or Subgroup (eg, Steering Committee)		
Katharina	Klatte		MSc	Clinical Trial Unit, Department of Clinical Research, University of Basel and University Hospital Basel	Basel, Switzerland	Systematically searched for the trials in clinical trial registries and extracted the trial characteristics, critically revised the manuscript and approved the final version.			
Dmitry	Gryaznov		MSc	Basel Institute for Clinical Epidemiology and Biostatistics, Department of Clinical Research, University Hospital Basel, University of Basel	Basel, Switzerland	Lead the ASPIRE study group to extract trial characteristics from the ethically approved study protocols, critically revised the manuscript and approved the final version.			
Ala	Taji Heravi		MSc	Basel Institute for Clinical Epidemiology and Biostatistics, Department of Clinical Research, University Hospital Basel, University of Basel	Basel, Switzerland	Systematically searched for the trials in clinical trial registries and extracted the trial characteristics, critically revised the manuscript and approved the final version.			

Supplemental Online Content: Nonauthor Collaborators

*Indicates required information. Only first name, last name, and suffix will appear in PubMed.

*First Name and Mi	*Last Name	*Suffix (eg	Academic D	Institution	Location (city, state/province, country)	Role or Contribution, eg, chair, principal investigator	Group (if more than 1 Group listed in the byline) and/or Subgroup (eg, Steering Committee)		
Nilabh	Ghosh		MSc	Department of Neurosurgery and Department of Biomedicine, University Hospital Basel, University of Basel	Basel, Switzerland	Systematically searched for the trials in clinical trial registries and extracted the trial characteristics, critically revised the manuscript and approved the final version.			
Ioana R	Marian		MSc	Oxford Clinical Trials Research Unit, Centre for Statistics in Medicine, Nuffield Department of Orthopaedics, Rheumatology and Musculoskeletal Sciences, University of Oxford	Oxford, UK	Systematically searched for the trials in clinical trial registries and extracted the trial characteristics, critically revised the manuscript and approved the final version.			
Hopin	Lee		PhD	Centre for Statistics in Medicine, Nuffield Department of Orthopaedics, Rheumatology and Musculoskeletal Sciences, University of Oxford	Oxford, UK	Systematically searched for the trials in clinical trial registries and extracted the trial characteristics, critically revised the manuscript and approved the final version.			

Supplemental Online Content: Nonauthor Collaborators

*Indicates required information. Only first name, last name, and suffix will appear in PubMed.

***First Name and Mi**	***Last Name**	***Suffix (eg**	Academic [	Institution	Location (city, state/province, country)	Role or Contribution, eg, chair, principal investigator	Group (if more than 1 Group listed in the byline) and/or Subgroup (eg, Steering Committee)		
Anita	Mansouri		MSc	Oxford Clinical Trials Research Unit, Centre for Statistics in Medicine, Nuffield Department of Orthopaedics, Rheumatology and Musculoskeletal Sciences, University of Oxford	Oxford, UK	Systematically searched for the trials in clinical trial registries and extracted the trial characteristics, critically revised the manuscript and approved the final version.			
Szimonetta	Lohner		MD, PhD	Cochrane Hungary, Clinical Centre of the University of Pécs, Medical School, University of Pécs	Pécs, Hungary	Systematically searched for the trials in clinical trial registries and extracted the trial characteristics, critically revised the manuscript and approved the final version.			
Ramon	Saccilotto		MD	Clinical Trial Unit, Department of Clinical Research, University of Basel and University Hospital Basel	Basel, Switzerland	Lead the ASPIRE study group to extract trial characteristics from the ethically approved study protocols, critically revised the manuscript and approved the final version.			

Supplemental Online Content: Nonauthor Collaborators

*Indicates required information. Only first name, last name, and suffix will appear in PubMed.

*First Name and Mi	*Last Name	*Suffix (eg	Academic D	Institution	Location (city, state/province, country)	Role or Contribution, eg, chair, principal investigator	Group (if more than 1 Group listed in the byline) and/or Subgroup (eg, Steering Committee)		
Edris	Nury		MSc	Institute for Evidence in Medicine (for Cochrane Germany Foundation), Faculty of Medicine and Medical Center, University of Freiburg	Freiburg, Germany	Systematically searched for the trials in clinical trial registries and extracted the trial characteristics, critically revised the manuscript and approved the final version.			
An-Wen	Chan		MD, Dphil	Department of Medicine, Women's College Research Institute, Women's College Hospital, University of Toronto	Toronto, Ontario, Canada	Established contacts with representatives from trial registries, conducted interviews with representatives from trial registries, critically revised the manuscript and approved the final version.			
Anette	Blümle		PhD	Institute for Evidence in Medicine (for Cochrane Germany Foundation), Faculty of Medicine and Medical Center, University of Freiburg	Freiburg, Germany	Systematically searched for the trials in clinical trial registries and extracted the trial characteristics, critically revised the manuscript and approved the final version.			

Supplemental Online Content: Nonauthor Collaborators

*Indicates required information. Only first name, last name, and suffix will appear in PubMed.

*First Name and Mi	*Last Name	*Suffix (eg	Academic D	Institution	Location (city, state/province, country)	Role or Contribution, eg, chair, principal investigator	Group (if more than 1 Group listed in the byline) and/or Subgroup (eg, Steering Committee)		
Ayodele	Odutayo		MD, Dphil	Centre for Statistics in Medicine, Nuffield Department of Orthopaedics, Rheumatology and Musculoskeletal Sciences, University of Oxford	Oxford, UK	Set-up the data extraction platform, critically revised the manuscript and approved the final version.			
Sally	Hopewell		Dphil	Centre for Statistics in Medicine, Nuffield Department of Orthopaedics, Rheumatology and Musculoskeletal Sciences, University of Oxford	Oxford, UK	Designed the reliability study, critically revised the manuscript and approved the final version.			
Matthias	Briel		MD, PhD	Basel Institute for Clinical Epidemiology and Biostatistics, Department of Clinical Research, University Hospital Basel, University of Basel	Basel, Switzerland	Designed the reliability study, conducted interviews with representatives from trial registries, critically revised the manuscript and approved the final version.			
Belinda	von Niederhäusern		PhD	Department of Clinical Research, Clinical Trial Unit, University Hospital Basel and University of Basel	Basel, Switzerland	Assessing ethically approved study protocols and extracting data			
Benjamin	Kasenda		MD PhD	Basel Institute for Clinical Epidemiology and Biostatistics, Department of Clinical Research, University Hospital Basel, University of Basel	Basel, Switzerland	Assessing ethically approved study protocols and extracting data			

Supplemental Online Content: Nonauthor Collaborators

*Indicates required information. Only first name, last name, and suffix will appear in PubMed.

*First Name and Mi	*Last Name	*Suffix (eg	Academic [	Institution	Location (city, state/province, country)	Role or Contribution, eg, chair, principal investigator	Group (if more than 1 Group listed in the byline) and/or Subgroup (eg, Steering Committee)		
Elena	Ojeda-Ruiz		MD	Basel Institute for Clinical Epidemiology and Biostatistics, Department of Clinical Research, University Hospital Basel, University of Basel	Basel, Switzerland	Assessing ethically approved study protocols and extracting data			
Stefan	Schandelmaier		MD PhD	Basel Institute for Clinical Epidemiology and Biostatistics, Department of Clinical Research, University Hospital Basel, University of Basel	Basel, Switzerland	Assessing ethically approved study protocols and extracting data			
Dominik	Mertz		MD MSc	Department of Health Research Methods, Evidence, and Impact, McMaster University	Hamilton, Canada	Assessing ethically approved study protocols and extracting data			
Yuki	Tomonaga		PhD	Epidemiology, Biostatistics and Prevention Institute, University of Zurich	Zurich, Switzerland	Assessing ethically approved study protocols and extracting data			
Alain	Amstutz		MD	Basel Institute for Clinical Epidemiology and Biostatistics, Department of Clinical Research, University Hospital Basel, University of Basel	Basel, Switzerland	Assessing ethically approved study protocols and extracting data			
Christiane	Pauli-Magnus		MD	Department of Clinical Research, Clinical Trial Unit, University Hospital Basel and University of Basel	Basel, Switzerland	Assessing ethically approved study protocols and extracting data			
Constantin	Sluka		PhD	Department of Clinical Research, Clinical Trial Unit, University Hospital Basel and University of Basel	Basel, Switzerland	Assessing ethically approved study protocols and extracting data			
Karin	Bischoff		MSc	Institute for Evidence in Medicine, Medical Center – University of Freiburg, Faculty of Medicine, University of Freiburg	Freiburg, Germany	Assessing ethically approved study protocols and extracting data			

Supplemental Online Content: Nonauthor Collaborators

*Indicates required information. Only first name, last name, and suffix will appear in PubMed.

*First Name and Mi	*Last Name	*Suffix (eg	Academic D	Institution	Location (city, state/province, country)	Role or Contribution, eg, chair, principal investigator	Group (if more than 1 Group listed in the byline) and/or Subgroup (eg, Steering Committee)		
Katharina	Wollmann		MSc	Institute for Evidence in Medicine, Medical Center – University of Freiburg, Faculty of Medicine, University of Freiburg	Freiburg, Germany	Assessing ethically approved study protocols and extracting data			
Laura	Rehner		PhD	Institute for Evidence in Medicine, Medical Center – University of Freiburg, Faculty of Medicine, University of Freiburg,	Freiburg, Germany	Assessing ethically approved study protocols and extracting data			
Joerg J.	Meerpohl		MD	Institute for Evidence in Medicine, Medical Center – University of Freiburg, Faculty of Medicine, University of Freiburg,	Freiburg, Germany	Assessing ethically approved study protocols and extracting data			
Alain	Nordmann		MD MSc	Basel Institute for Clinical Epidemiology and Biostatistics, Department of Clinical Research, University Hospital Basel, University of Basel	Basel, Switzerland	Assessing ethically approved study protocols and extracting data			
Jacqueline	Wong		MD MSc	Department of Health Research Methods, Evidence, and Impact, McMaster University	Hamilton, Canada	Assessing ethically approved study protocols and extracting data			
Ngai	Chow		BSc	Department of Health Research Methods, Evidence, and Impact, McMaster University	Hamilton, Canada	Assessing ethically approved study protocols and extracting data			
Patrick Jiho	Hong		MD	Department of Health Research Methods, Evidence, and Impact, McMaster University	Hamilton, Canada	Assessing ethically approved study protocols and extracting data			

*Indicates required information. Only first name, last name, and suffix will appear in PubMed.

*First Name and Mi	*Last Name	*Suffix (eg	Academic D	Institution	Location (city, state/province, country)	Role or Contribution, eg, chair, principal investigator	Group (if more than 1 Group listed in the byline) and/or Subgroup (eg, Steering Committee)		
Kimberly	Mc Cord		PhD	Basel Institute for Clinical Epidemiology and Biostatistics, Department of Clinical Research, University Hospital Basel, University of Basel	Basel, Switzerland	Assessing ethically approved study protocols and extracting data			
Sirintip	Sricharoenchai		MD MSc	Basel Institute for Clinical Epidemiology and Biostatistics, Department of Clinical Research, University Hospital Basel, University of Basel	Basel, Switzerland	Assessing ethically approved study protocols and extracting data			
Jason W.	Busse		PhD	Department of Health Research Methods, Evidence, and Impact, McMaster University	Hamilton, Canada	Assessing ethically approved study protocols and extracting data			
Arnav	Agarwal		MD	Department of Health Research Methods, Evidence, and Impact, McMaster University	Hamilton, Canada	Assessing ethically approved study protocols and extracting data			
Matthias	Schwenkglenks		PhD MPH	Epidemiology, Biostatistics and Prevention Institute, University of Zurich	Zurich, Switzerland	Assessing ethically approved study protocols and extracting data			
Giusi	Moffa		PhD	Basel Institute for Clinical Epidemiology and Biostatistics, Department of Clinical Research, University Hospital Basel, University of Basel	Basel, Switzerland	Assessing ethically approved study protocols and extracting data			
Lars G.	Hemkens		MD MPH	Basel Institute for Clinical Epidemiology and Biostatistics, Department of Clinical Research, University Hospital Basel, University of Basel	Basel, Switzerland	Assessing ethically approved study protocols and extracting data			
Erik	von Elm		MD MPH	Cochrane Switzerland, Centre for Primary Care and Public Health (Unisanté), University of Lausanne	Lausanne, Switzerland	Assessing ethically approved study protocols and extracting data			
